# CFTR dysfunction increases endoglin and TGF‐*β* signaling in airway epithelia

**DOI:** 10.14814/phy2.13977

**Published:** 2019-02-25

**Authors:** Teodora Nicola, Farruk L. Kabir, Tatjana Coric, Stephanie B. Wall, Weifeng Zhang, Masheika James, Mark MacEwen, Changchun Ren, Brian Halloran, Namasivayam Ambalavanan, William T. Harris

**Affiliations:** ^1^ Division of Neonatology Department of Pediatrics University of Alabama at Birmingham Birmingham Alabama; ^2^ Division of Pediatric Pulmonology Department of Pediatrics University of Alabama at Birmingham Birmingham Alabama; ^3^ Department of Pharmacology and Toxicology University of Alabama at Birmingham Birmingham Alabama; ^4^ Gregory Fleming James Cystic Fibrosis Center University of Alabama at Birmingham Birmingham Alabama

**Keywords:** CFTR, cystic fibrosis, Endoglin, lung disease, TGF‐beta

## Abstract

Endoglin (ENG) regulates signaling by transforming growth factor‐*β* (TGF‐*β*), a genetic modifier of cystic fibrosis (CF) lung disease severity. We hypothesized that ENG mediates TGF‐*β* pathobiology in CF airway epithelia. Comparing CF and non‐CF human lungs, we measured ENG by qPCR, immunoblotting and ELISA. In human bronchial epithelial cell lines (16HBE), we used CFTR siRNA knockdown and functional inhibition (CFTR_INH_‐172) to connect loss of CFTR to ENG synthesis. Plasmid overexpression of ENG assessed the direct effect of ENG on TGF‐*β* transcription and signal amplification in 16HBE cells. We found ENG protein to be increased more than fivefold both in human CF bronchoalveolar fluid (BALF) and human CF lung homogenates. ENG transcripts were increased threefold in CF, with a twofold increase in TGF‐*β* signaling. CFTR knockdown in 16HBE cells tripled ENG transcription and doubled protein levels with corresponding increases in TGF‐*β* signaling. Plasmid overexpression of ENG alone nearly doubled TGF‐*β*1 mRNA and increased TGF‐*β* signaling in 16HBE cells. These experiments identify that loss of CFTR function increases ENG expression in CF epithelia and amplifies TGF‐*β* signaling. Targeting ENG may offer a novel therapeutic opportunity to address TGF‐*β* associated pathobiology in CF.

## Introduction

Cystic Fibrosis (CF) is due to genetic mutations in CFTR that impede chloride and bicarbonate transport in airway epithelia (Rowe et al. 2005). CF lung disease is characterized by chronic infection and inflammation that ultimately leads to respiratory failure (Stoltz et al. 2015). Disease progression in CF is quite heterogeneous, with transforming growth factor‐beta (TGF‐*β*) being a major gene modifier of disease severity (Arkwright et al. 2000; Drumm et al. 2005; Bremer et al. 2008; Collaco and Cutting 2008; Collaco et al. 2008). TGF‐*β*_1_ genetic polymorphisms are associated with a more rapid pulmonary decline (Arkwright et al. 2000), with an odds ratio >2 of having severe lung disease(Drumm et al. 2005) and an amplified deterioration (>20% decline) in lung function after second hand smoke exposure (Collaco et al. 2008). TGF‐*β* is a pleiotropic cytokine with effects on lung development, immune modulation, and fibrotic response (Massague 2012).

We have previously reported that increased TGF‐*β* signaling is a major contributor to lung fibrosis in CF (Harris et al. 2013), with increased plasma and bronchoalveolar fluid (BALF) TGF‐*β* concentrations in subjects with more advanced lung disease (Harris et al. 2009, 2011). In addition to the effects of increased TGF‐*β* signaling on lung fibrosis and remodeling, TGF‐*β* has direct suppressive actions on CFTR expression in primary differentiated human bronchial epithelial cells (Snodgrass et al. 2013; Sun et al. 2014), with antagonism of recently approved CFTR modulator therapy (Lutful Kabir et al. 2018).

As TGF‐*β* is fundamental to normal growth, development and immunomodulation, addressing excessive TGF‐*β* signaling must be nuanced, preferably utilizing available regulatory pathways that may become disturbed in chronic disease states. Multiple cells produce TGF‐*β* in the lung. Within the airway, macrophages and airway epithelia are likely the main contributors, with fibroblasts and other inflammatory cells contributing to TGF‐b production in the parenchyma. For TGF‐*β* in disease, dysregulated activation may be more pathogenic than the production sources. In the CF context, dysregulated activation may be secondary to increased lung inflammation, proteases, altered pH, and mechanical strain (Annes et al. 2003; Shi et al. 2011; Hinz 2015). Endoglin (ENG) offers an appealing endogenous regulatory pathway that may be utilized to normalize TGF‐*β* signaling in CF lungs. ENG is a 180 kDa homodimer cell surface glycoprotein (a TGF‐*β* Type III co‐receptor) that binds to TGF ‐*β*1, ‐*β*3, activin A and BMP‐2,‐7. Central to TGF‐*β* signaling, ENG has previously been identified on fibroblasts, activated macrophages, endothelial cells, and smooth muscle cells (Conley et al. 2000). Two different isoforms, L‐endoglin (full length) and S‐endoglin (short) differing in the amino acid composition of their cytoplasmic tails(Rodriguez‐Pena et al. 2001, 2002; Prieto et al. 2005; Velasco et al. 2008) share the capacity to bind TGF‐*β*. Recently, endoglin has been identified as a biomarker of CF liver disease (Rath et al. 2013).

We hypothesized that endoglin may mediate increased TGF‐*β* signaling in CF epithelia. The results of our study suggest ENG may contribute to CF respiratory disease and offer a possible therapeutic target to disrupt pathogenic TGF‐*β* sequelae in CF lungs.

## Methods

### Institutional approval

University of Alabama at Birmingham (UAB) Institutional Review Board approval (Protocol # X081204008 and #F070813009) was obtained prior to conducting these studies.

### Immunohistochemistry

Formalin‐fixed, paraffin‐embedded blocks were sectioned at 10 *μ*m. Sections of parenchyma were taken from the periphery of each lobe to control for regional heterogeneity. Heat‐induced epitope retrieval with 0.02 mol/L of citrate buffer (pH 6.0) at 97°C for 20 min was performed and immunohistochemistry accomplished with DAKO EnVision kit (cat.# Rabbit K4010). Rabbit anti‐human ENG antibody (SC‐20632, 1∶50 dilution, Santa Cruz Biotechnology) was used for detection by diaminobenzine tetrachloride (DAB). Negative control slides (lacking primary antibody) were prepared in all cases.

### Quantitative real‐time PCR (qPCR)

SYBR Green RT‐PCR kit (Applied Biosystems) was used in the Bio‐Rad iCycler System with the following human primer (3′‐5′): ENG forward CGTGGACAGCATGGACC, ENG reverse GATGCAGGAAGACACTGCTG and 18S, TGF*β*1 and PAI‐1 as previously described (Ambalavanan et al. 2008a,2008b; Nicola et al. 2009, 2011; Olave et al. 2012).

GAPDH and actin were also used as internal qPCR controls.

### Immunoblotting

Primary rabbit antibodies pSmad2, total SMAD2 (Millipore), *β*‐tubulin (Sigma‐Aldrich), *β*‐actin (Millipore), CFTR (Cell Signaling and Millipore) and Endoglin (Boster #PA1395) were used at 1:2000 dilutions. TGF‐*β* signaling was measured by phosphorylation of Smad2 (the major TGF‐*β* signaling pathway) relative to total SMAD. Endoglin was normalized to *β*‐tubulin.

### Bronchoalveolar Lavage Sample and Lung Tissue Preparations

Remnant pediatric CF (*n* = 14) and non‐CF (*n* = 5) bronchoalveolar lavage (BAL) fluid was obtained at the time of clinically indicated flexible bronchoscopy utilizing standard techniques (Harris et al. 2009; Peterson‐Carmichael et al. 2009). For comparison, BALF ENG was also measured in non‐CF, nonbronchiectatic children undergoing clinically indicated bronchoscopy for the evaluation of either chronic/recurrent wheeze or recurrent pneumonia. After centrifugation (Harris et al. 2009), the BAL supernatant was used to quantify ENG protein levels by ELISA. Explanted CF human lung tissues were obtained at the time of transplant (CF, *n*  =  10) and/or failed lung transplant donors (non‐CF, *n*  =  10). Whole lung homogenates were subject to immunoblotting.

### Cell culture

We utilized the 16HBE14o‐ (16HBE) cell line that models bronchial epithelial cell phenotype (CFTR expression, transepithelial resistance, tight‐junctions, and ciliation) to model the association between CFTR and ENG in airway epithelia. Unless otherwise specified results were from at least three separate experiments performed in triplicate. The full‐length ENG Plasmid DNA (pCD105) was generously provided by Dr. Calvin PH Vary (Main Medical Research Institute, Scarborough, Main) (Liu et al. 2002; Breen et al. 2013). Cells transfected with the pcDNA3.1 plasmid are included as a negative control (vector control).

### CFTR knockdown in 16HBE cells

Downregulation of CFTR in 16HBE cells was performed utilizing siRNA knockdown techniques as previously described (Dalby et al. 2004). CFTR siRNA (Thermo Fisher, Cat#4392420‐s2945, ‐s2946, ‐s2947) and scrambled siRNA was used as a negative control (Cat# 4390843). Current data shows CFTR siRNA (Cat#4392420‐s2945) as a representative. CFTR siRNA knockdown lowered CFTR expression by 70‐80%.

### CFTR functional inhibition

CFTR functional inhibition was performed with 20 *μ*mol/L CFTR_INH_‐172 (Sigma, Cat#C2992‐5MG), a thiazolidinone that selectively blocks the CFTR channel (K_i_= 300 nmol/L) in a voltage‐independent manner (Ma et al. 2002; Taddei et al. 2004). CFTR_INH_‐172 directly modulates chloride gating at the channel and does not prevent elevation of cAMP or inhibit other pumps or channels (Peterson‐Carmichael et al. 2009; Olave et al. 2012).

### ELISA to quantify ENG protein

Quantitative analysis of ENG levels was measured in BAL fluid of CF and non‐CF subjects by following manufacturer's protocol using the human CD105 ELISA kit (Boster Biological Technology, Cat#EK0644). BALF was centrifuged at 500 g 5 min with the cell‐free supernatant stored at −80°C for subsequent assays. The standard product used in this kit is extracellular part of recombinant human CD105, from E26 to G586. Previously, we published increased TGF‐*β*1 in BAL fluid from children with cystic fibrosis (Harris et al. 2009; Lutful Kabir et al. 2018).

### TGF‐β1 production

Protein levels of TGF‐*β* were measured in cell culture studies using a mink lung cell bioassay (Abe et al. 1994).

### Statistics

Parametric data was analyzed by t‐test for comparison of two variables, and ANOVA with Tukey–Kramer posttest analysis for multiple comparisons. Analysis of nonparametric data utilized the Mann–Whitney test. For all analytical studies, significance was assigned to *P* ≤ 0.05.

## Results

### In vivo

#### ENG is increased in CF lung

##### Bronchoalveolar lavage (BAL) fluid

**Figure 1:**
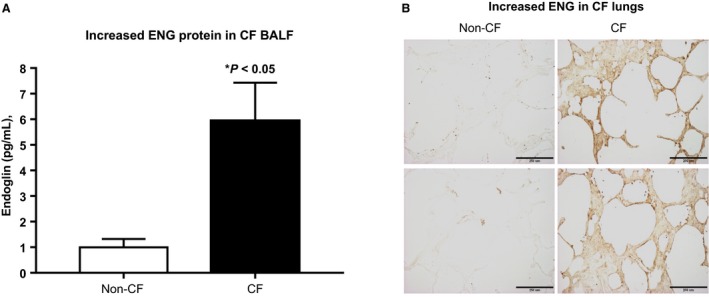
Increased endoglin in CF bronchoalveolar lavage fluid (BAL) fluid. Quantitative analysis by human CD105 ELISA kit (Cat.# EK0644, Boster Immuno‐Leader Biotechnology) indicates a fivefold increase in endoglin in severe CF (*n* = 14) compared to non‐CF samples (*P < 0.05, *n* = 5; **A**). Immunohistochemistry on paraffin‐embedded lung sections shows increased ENG (brown staining) in CF lungs with severe disease as compared to non‐ CF (100x; primary Ab: Rabbit polyclonal anti‐human ENG, 1:50 dilution, Santa Cruz, Calibration bar = 250 *μ*m) (B).

Remnant BAL fluid was obtained in CF (mean age 8.7 years, FEV1 78%, 35% Pseudomonas positive) and non‐CF controls for ENG quantification. ENG was increased more than fivefold in CF BAL fluid compared to the non‐CF sample (ENG mean±SEM, CF: 5.935 ± 1.493, *n* = 14 vs. non‐CF: 1 ± 0.3233, *n* = 5; *P* < 0.05; Fig.**** 1 **A**). Immunohistochemistry for ENG on paraffin‐embedded lung sections showed increased staining (brown) in CF disease as compared to non‐CF lung (Fig.**** 1 **B)**.

##### Lung homogenates

**Figure 2:**
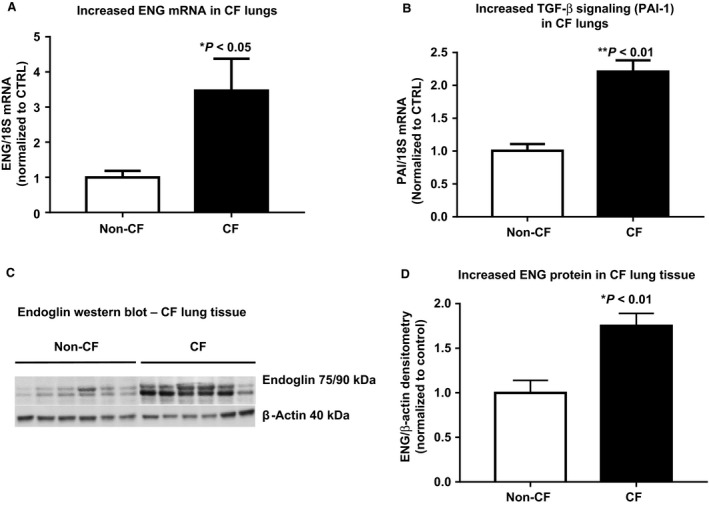
Increased endoglin and TGF‐*β* signaling in CF lungs. (A) ENG (threefold**,** * *P* < 0.05) and (B) PAI‐1 mRNA (twofold, ** *P* < 0.01) are increased in human lung homogenates of CF (*n* = 5) compared to non‐CF lung tissue (*n* = 5) by (A–B) qPCR and (C–D) immunoblotting (ninefold, C–D, * *P* < 0.01) normalized to *β*‐Actin.

Lung tissue was also obtained from CF subjects (explanted end‐stage lung tissue obtained at the time of lung transplant, *n* = 5, mean age (±SEM) 24 ± 2.0 years) and non‐CF controls (failed tissue donor, *n* = 5, mean age 35 ± 6.5 years). ***Real time qPCR*** revealed a threefold increase in ENG mRNA (CF 3.5 ± 1.8 vs. non‐CF: 1.0 ± 0.4, *n* = 5; *P* < 0.05; Fig.**** 2 **A**), a twofold increase in the representative TGF‐*β* signal PAI‐1 mRNA (CF 2.2 ± 0.3 vs. non‐CF: 1.0 ± 0.2, *n* = 5; *P* < 0.01; Fig. 2B) and a 1.5‐fold increase in TGF‐*β*1 mRNA (CF 1.5 ± 0.3 vs. non‐CF: 1.0 ± 0.3, *n* = 5; *P* < 0.05, data not shown) in lung homogenates of CF compared to non‐CF lung indicating that ENG and TGF‐*β* signaling (PAI‐1) are increased at the transcription level.

##### Immunoblotting

This data from lung homogenates shows a twofold increase in ENG protein levels in severely diseased CF lungs compared to normal lung (ENG/*β*‐actin ratio: CF 1.75 ± 0.1403, *n* = 6 vs. non‐CF: 1 ± 0.1385, *n* = 6; *P* < 0.01). These findings indicate that ENG protein is increased in diseased CF tissue (Fig. 2C–D).

### In vitro

#### Effect of CFTR manipulation on ENG and TGF*β* synthesis

**Figure 3:**
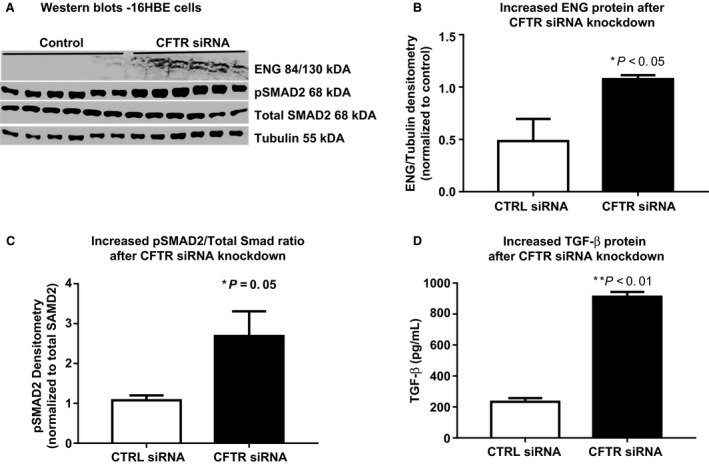
CFTR knockdown increases endoglin and TGF‐*β* in airway epithelia. CFTR siRNA knockdown in 16HBE bronchial epithelial cells increases (A) immunoblotting for (B) ENG (twofold, * *P* < 0.05, *n* = 6) and (**C**) phosphorylated Smad2 (pSmad2, a marker of TGF‐*β* signaling) 1.09 ± 0.07 versus 0.80 ± 0.05, *n* = 6, ** *P* < 0.01) quantified by densitometry with (D) TGF‐*β* protein levels in cultured media increased fourfold (*P* < 0.01, *n* = 6) quantified by bioassay.

To elucidate the relationship between CFTR dysfunction, and endoglin‐associated TGF‐*β* signaling, we utilized CFTR siRNA to knockdown CFTR in bronchial epithelial cells (16HBE cell line). CFTR siRNA knockdown doubled both endoglin protein levels (CFTR siRNA 1.07 ± 0.02 vs. Sham siRNA 0.47 ± 0.2, *n* = 6; *P* < 0.05, Fig. 3B) and Smad2 phosphorylation (the canonical signaling pathway for TGF‐*β*)(Derynck and Zhang 2003) in airway epithelial cells (CFTR siRNA 2.688 ± 0.6189, *n* = 6, Sham siRNA 1.078 ± 0.1236, *n* = 6; *P* = 0.0502, Fig. 3C). Similarly, TGF‐*β* protein levels were increased more than fourfold (CFTR siRNA: 912 ± 31.3 pg/mL vs. Sham siRNA: 234 ± 23 pg/mL, *n* = 6; *P* < 0.005, Fig. 3D) in cultured media as measured by standard bioassay (Abe et al. 1994). Our CFTR siRNA knockdown efficiency was 70–80%.

**Figure 4:**
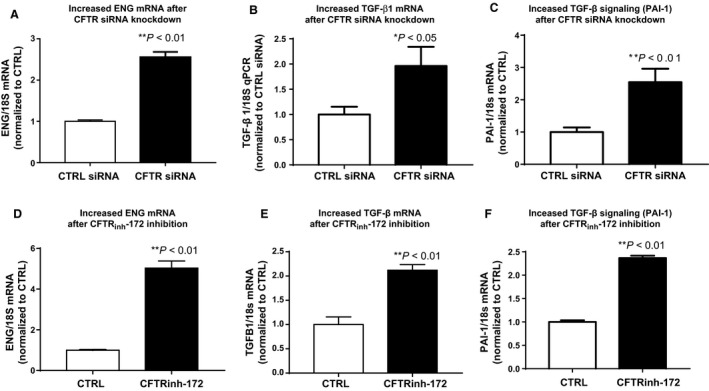
Loss of CFTR by siRNA knockdown or by functional CFTRinh‐172 inhibition increases endoglin and TGF *β* signaling. CFTR siRNA knockdown increases (A) ENG mRNA twofold (**P < 0.01, *n* = 6), (B) TGF *β*1 mRNA twofold (*P < 0.05, *n* = 5) and (C) PAI‐1 mRNA, a representative marker of TGF‐*β* signaling (**P < 0.01, *n* = 5) compared to control (CTRL). Functional inhibition of CFTR with CFTR_inh_‐172 increases (D) ENG mRNA fivefold (**P < 0.01, *n* = 4) and doubles both (E) TGF *β*1 mRNA (**P < 0.01, *n* = 4) and (F) PAI‐1 mRNA (**P < 0.01, *n* = 3) compared to control (CTRL).

Loss of CFTR by siRNA knockdown in 16HBE cells also increased ENG mRNA threefold (CFTR siRNA 2.73 ± 0.17 vs. Sham siRNA 1.00 ± 0.04, *n* = 6; *P* < 0.01, Fig.**** 4 **A**), increased TGF‐*β*1 mRNA 2‐fold (CFTR siRNA 1.96 ± 0.84 vs. Sham siRNA 1.00 ± 0.37, *n* = 6; *P* < 0.5, Fig.**** 4 **B**) and TGF‐*β* signaling 2.5‐fold (PAI‐1 mRNA, CFTR siRNA 2.54 ± 0.9 vs. Sham siRNA 1.00 ± 0.31, *n* = 6; *P* < 0.01, Fig.**** 4 **C**) compared to control cells.

**Figure 5:**
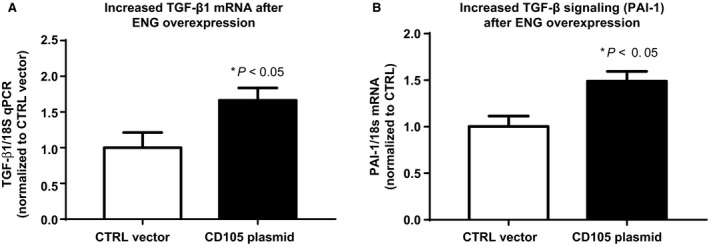
Endoglin plasmid overexpression increases TGF‐*β* transcription and signaling. Overexpression of ENG (pCD105 plasmid) in 16HBE bronchial epithelial cells significantly increases (A) TGF‐*β*1 mRNA (*P < 0.05) and (B) TGF‐*β* signaling (PAI‐1 mRNA, * *P* < 0.05, *n* = 6) compared to vector control (CTRL).

In addition to siRNA knockdown, functional inhibition of CFTR was achieved with 20 *μ*mol/L CFTR_INH_‐172 in 16HBE cells. Loss of CFTR function resulted in a fivefold increase in ENG mRNA (Control 16HBE 1 ± 0.04 vs. CFTR_INH_‐172: 5.02 ± 0.85, *n* = 6; *P* < 0.01, Fig. 4D), a twofold increase in TGF‐*β*1 mRNA (Control 16HBE 1 ± 0.31 vs. CFTR_INH_‐172: 2.21 ± 0.23, *n* = 6; *P* < 0.01, Fig. 5E) and TGF‐*β* signaling 2.5‐fold increase (PAI‐1; Control 16HBE 1 ± 0.06 vs. CFTR_INH_‐172: 2.37 ± 0.08, *n* = 6; *P* < 0.01, Fig. 4F) compared to control cells, suggesting a direct impact of CFTR dysfunction on ENG and TGF‐*β*1 transcription.

#### Effect of ENG overexpression on TGF‐*β* signaling

The previous studies showed that loss of CFTR function increased ENG expression with corresponding increase in TGF‐*β*1 transcription and signaling. We then wanted to evaluate the effect of ENG overexpression on TGF‐*β*1 signaling. To test the hypothesis that upregulated ENG increased TGF‐*β*1 signaling in airway epithelia, we overexpressed ENG by plasmid‐DNA transfection. In 16HBE cells, ENG overexpression increased TGF‐*β*1 mRNA (control 1 ± 0.43; 1.67 ± 0.38, *n* = 6; *P* < 0.05, Fig. 5A) and TGF‐*β* signaling (PAI‐1; 1 ± 0.27; 1.5 ± 0.23, n = 6; *P* < 0.05, Fig. 5B) showing a direct relationship between ENG stimulation and TGF‐*β*1 signaling in airway epithelia.

Our data suggest a relationship between CFTR, ENG and TGF‐*β*1. Loss of CFTR increases both ENG and TGF‐*β*1, with increased TGF‐*β*1 further potentiating TGF‐*β*1 signaling.

## Discussion

This study is the first to show that endoglin is increased in CF lungs and that loss of CFTR directly stimulates endoglin production in CF epithelia. Increases in endoglin (either from loss of CFTR or after plasmid overexpression) amplifies TGF‐*β* signaling, demonstrating a potentially modifiable pathway to ameliorate TGF‐*β* pathobiology in CF tissue. Marked Elevations of endoglin in end‐stage CF lung specimens underscores the importance of endoglin to tissue fibrosis, and suggests a contribution of endoglin to CF lung disease progression.

Our study has several significant strengths. First, we have utilized multiple in vivo measures to indicate endoglin upregulation in CF lungs. Quantitative PCR, immunoblotting of lung homogenate, ENG immunohistochemistry, and ELISA of BAL fluid all demonstrate increased endoglin in CF pulmonary disease. Circulating endoglin levels have recently been identified as a biomarker of CF liver disease (Rath et al. 2013), and our findings suggest endoglin may also be significantly higher in CF respiratory samples.

Second, we have utilized in vitro studies to demonstrate that specific loss of CFTR, by siRNA knockdown or by CFTR_INH_‐172, significantly increases endoglin transcription and protein levels in bronchial epithelia, with corresponding upregulation of TGF‐*β* synthesis and signaling. Potential limitations of siRNA are off‐target effects, but to eliminate the possibility that the phenotype we see is due to off‐target effects we used: (1) three different siRNA targeting CFTR, (2) siRNAs are chemically modified to eliminate off‐target effects of the passenger strand, and (3) we verified the effects by using CFTR_INH_‐172. Similarly, we acknowledge that CFTR inhibitors could exert nonspecific effects regarding ROS production, mitochondrial failure, and activation of the NF‐*κ*B signaling pathway, independently of CFTR inhibition (Kelly et al. 2010; Melis et al. 2014). However, the fact that the siRNAs and inhibitor have the same effect shows that the effects are specific to CFTR, and not due to possible nonspecific action of the inhibitor.

We have previously identified an inhibitory effect of exogenous TGF‐*β* on CFTR function (Sun et al. 2014; Lutful Kabir et al. 2018), but these are the first data to identify the feedback relationships in the opposite direction (e.g. that loss of CFTR increases TGF‐*β* signaling with endoglin possibly mediating this interaction).

Additionally, we have utilized an ENG overexpressing construct to demonstrate the direct effect of increased endoglin on TGF‐*β* signaling in airway epithelia. These data suggest an interlinked pathologic triad in CF whereby loss of CFTR increases endoglin production which further potentiates profibrotic TGF‐*β* signaling. Future studies will determine the potential of endoglin inhibition to interrupt the feed–forward relationship between CFTR dysfunction and exuberant TGF‐*β* signaling in primary airway epithelia.

Endoglin invokes discrete TGF‐*β* responses across cell types, prompting this investigation of endoglin‐mediated effects in airway epithelia. Pharmaceutical agents are currently under development to manipulate endoglin as a TGF‐*β* “brake” that may have potential application to CF. The most established of these is TRC105, an endoglin antibody that has been studied for the treatment of multiple solid tumor types, including soft tissue sarcoma, renal cell carcinoma, glioblastoma, hepatocellular carcinoma and colorectal cancer (Rosen et al. 2012, 2014; Gordon et al. 2014; Paauwe et al. 2016). TRC105 has generally been well tolerated as a single agent and in combination with VEGF inhibitors in these clinical oncology trials, but safety (and efficacy) would obviously need to be evaluated in preclinical CF model systems.

Previously, we have explored the mechanisms of the TGF‐*β*/CFTR axis in the forward direction, in which miRNA mediate this interaction (Lutful Kabir et al. 2018). We have specifically identified that miR‐145 mediates TGF‐*β* suppression of CFTR synthesis and function. TGF‐*β* stimulates miR‐145 expression and miR‐145 has a specific binding site on the 3′‐untranslated region of CFTR to limit transcript stability and protein translation. miRNA may similarly regulate the feedback relationship whereby loss of CFTR alters levels of miRNA that regulate endoglin expression.

We acknowledge the limitations of our study that utilizes manipulated cell culture models, tissue from end‐stage explanted lung tissue, and bronchoalveolar lavage fluid from children with relatively mild disease. To address this, we have utilized multiple specimens and biochemical tools to ascertain the relationship of endoglin to TGF‐*β* in CF cells. Although we have shown increased endoglin and TGF‐*β* signaling in lung tissue, and manipulated both CFTR and endoglin in airway epithelia to isolate direct interactions in epithelial model systems, we acknowledge the complexity of the CF microenvironment and the multiple triggers for increased TGF‐*β* signaling in the disease.

Although we have attempted to demonstrate that loss of CFTR increases endoglin expression that potentiates TGF‐*β* signaling in CF epithelia, more definitive delineation of a pathogenic contribution of endoglin to respiratory decline may require a manipulated animal model. We also acknowledge the complexity of the regulation of TGF‐*β* signaling to CF disease. While TGF‐*β* is a genetic modifier of disease progression and CFTR modulator response, TGF‐*β* is also a potent immunomodulatory that is essential to inflammatory resolution. Addressing TGF‐*β* pathobiology will require interruption of the extremes of TGF‐*β* signaling with a focus on bolstering regulatory pathways, while preserving normative function. We posit the current findings lay the foundation for considering endoglin inhibition as a nuanced mechanistic tool to dampen TGF‐*β* signaling in CF epithelia.

In summary, we are the first to report the association between endoglin and TGF‐*β* pathobiology in CF lung disease. We have investigated both human lung specimens and CF cell culture models to propose an interdependent relationship between loss of CFTR and endoglin upregulation that results in enhanced TGF‐*β* signaling in respiratory cells. Our results suggest that mechanistic evaluation and therapeutic consideration of endoglin inhibition in CF model systems merits further investigation.

## Conflict of Interest

None declared.

## References

[phy213977-bib-0001] Abe, M. , J. G. Harpel , C. N. Metz , I. Nunes , D. J. Loskutoff , and D. B. Rifkin . 1994 An assay for transforming growth factor‐beta using cells transfected with a plasminogen activator inhibitor‐1 promoter‐luciferase construct. Anal. Biochem. 216:276–284. 8179182 10.1006/abio.1994.1042

[phy213977-bib-0002] Ambalavanan, N. , T. Nicola , P. Li , et al. 2008a Role of matrix metalloproteinase‐2 in newborn mouse lungs under hypoxic conditions. Pediatr. Res. 63:26–32. 18043506 10.1203/PDR.0b013e31815b690d PMC2517580

[phy213977-bib-0003] Ambalavanan, N. , T. Nicola , J. Hagood , et al. 2008b Transforming growth factor‐beta signaling mediates hypoxia‐induced pulmonary arterial remodeling and inhibition of alveolar development in newborn mouse lung. Am. J. Physiol. Lung Cell. Mol. Physiol. 295:L86–L95. 18487357 10.1152/ajplung.00534.2007 PMC2494779

[phy213977-bib-0004] Annes, J. P. , J. S. Munger , and D. B. Rifkin . 2003 Making sense of latent TGFbeta activation. J. Cell Sci. 116(Pt 2):217–224. 12482908 10.1242/jcs.00229

[phy213977-bib-0005] Arkwright, P. D. , S. Laurie , M. Super , et al. 2000 TGF‐beta(1) genotype and accelerated decline in lung function of patients with cystic fibrosis. Thorax 55:459–462. 10817792 10.1136/thorax.55.6.459 PMC1745768

[phy213977-bib-0006] Breen, M. J. , D. M. Moran , W. Liu , X. Huang , C. P. Vary , and R. C. Bergan . 2013 Endoglin‐mediated suppression of prostate cancer invasion is regulated by activin and bone morphogenetic protein type II receptors. PLoS ONE 8:e72407. 23967299 10.1371/journal.pone.0072407 PMC3742533

[phy213977-bib-0007] Bremer, L. A. , S. M. Blackman , L. L. Vanscoy , et al. 2008 Interaction between a novel TGFB1 haplotype and CFTR genotype is associated with improved lung function in cystic fibrosis. Hum. Mol. Genet. 17:2228–2237. 18424453 10.1093/hmg/ddn123 PMC2902288

[phy213977-bib-0008] Collaco, J. M. , and G. R. Cutting . 2008 Update on gene modifiers in cystic fibrosis. Curr.Opin. Pulm. Med. 14:559–566. 18812833 10.1097/MCP.0b013e3283121cdc PMC2785460

[phy213977-bib-0009] Collaco, J. M. , L. Vanscoy , L. Bremer , et al. 2008 Interactions between secondhand smoke and genes that affect cystic fibrosis lung disease. JAMA 299:417–424. 18230779 10.1001/jama.299.4.417 PMC3139475

[phy213977-bib-0010] Conley, B. A. , J. D. Smith , M. Guerrero‐Esteo , C. Bernabeu , and C. P. Vary . 2000 Endoglin, a TGF‐beta receptor‐associated protein, is expressed by smooth muscle cells in human atherosclerotic plaques. Atherosclerosis 153:323–335. 11164421 10.1016/s0021-9150(00)00422-6

[phy213977-bib-0011] Dalby, B. , S. Cates , A. Harris , et al. 2004 Advanced transfection with Lipofectamine 2000 reagent: primary neurons, siRNA, and high‐throughput applications. Methods 33:95–103. 15121163 10.1016/j.ymeth.2003.11.023

[phy213977-bib-0012] Derynck, R. , and Y. E. Zhang . 2003 Smad‐dependent and Smad‐independent pathways in TGF‐beta family signalling. Nature 425:577–584. 14534577 10.1038/nature02006

[phy213977-bib-0013] Drumm, M. L. , M. W. Konstan , A. Handler , R. Pace , F. Zou , M. Zariwala , et al. 2005 Genetic modifiers of lung disease in cystic fibrosis. N. Engl. J. Med. 353:1443–1453. 16207846 10.1056/NEJMoa051469

[phy213977-bib-0014] Gordon, M. S. , F. Robert , D. Matei , D. S. Mendelson , J. W. Goldman , E. G. Chiorean , et al. 2014 An open‐label phase Ib dose‐escalation study of TRC105 (anti‐endoglin antibody) with bevacizumab in patients with advanced cancer. Clin. Cancer Res. 20:5918–5926. 25261556 10.1158/1078-0432.CCR-14-1143 PMC4570619

[phy213977-bib-0015] Harris, W. T. , M. S. Muhlebach , R. A. Oster , M. R. Knowles , and T. L. Noah . 2009 Transforming growth factor‐beta(1) in bronchoalveolar lavage fluid from children with cystic fibrosis. Pediatr. Pulmonol. 44:1057–1064. 19830844 10.1002/ppul.21079

[phy213977-bib-0016] Harris, W. T. , M. S. Muhlebach , R. A. Oster , M. R. Knowles , J. P. Clancy , and T. L. Noah . 2011 Plasma TGF‐beta(1) in pediatric cystic fibrosis: potential biomarker of lung disease and response to therapy. Pediatr. Pulmonol. 46:688–695. 21337732 10.1002/ppul.21430 PMC3115503

[phy213977-bib-0017] Harris, W. T. , D. R. Kelly , Y. Zhou , et al. 2013 Myofibroblast differentiation and enhanced TGF‐B signaling in cystic fibrosis lung disease. PLoS ONE 8:e70196. 23950911 10.1371/journal.pone.0070196 PMC3741283

[phy213977-bib-0018] Hinz, B. 2015 The extracellular matrix and transforming growth factor‐beta1: tale of a strained relationship. Matrix Biol. 47:54–65. 25960420 10.1016/j.matbio.2015.05.006

[phy213977-bib-0019] Kelly, M. , S. Trudel , F. Brouillard , F. Bouillaud , J. Colas , T. Nguyen‐Khoa , et al. 2010 Cystic fibrosis transmembrane regulator inhibitors CFTR(inh)‐172 and GlyH‐101 target mitochondrial functions, independently of chloride channel inhibition. J. Pharmacol. Exp. Ther. 333:60–69. 10.1124/jpet.109.162032. Epub 2010 Jan 5. 20051483

[phy213977-bib-0020] Liu, Y. , B. Jovanovic , M. Pins , C. Lee , and R. C. Bergan . 2002 Over expression of endoglin in human prostate cancer suppresses cell detachment, migration and invasion. Oncogene 21:8272–8281. 12447690 10.1038/sj.onc.1206117

[phy213977-bib-0021] Lutful Kabir, F. , N. Ambalavanan , G. Liu , P. Li , G. M. Solomon , C. V. Lal , et al. 2018 MicroRNA‐145 Antagonism Reverses TGF‐beta Inhibition of F508del CFTR Correction in Airway Epithelia. Am. J. Respir. Crit. Care Med. 197:632–643. 29232160 10.1164/rccm.201704-0732OC PMC6005236

[phy213977-bib-0022] Ma, T. , J. R. Thiagarajah , H. Yang , N. D. Sonawane , C. Folli , L. J. Galietta , et al. 2002 Thiazolidinone CFTR inhibitor identified by high‐throughput screening blocks cholera toxin‐induced intestinal fluid secretion. J. Clin. Investig. 110:1651–1658. 12464670 10.1172/JCI16112 PMC151633

[phy213977-bib-0023] Massague, J. 2012 TGFbeta signalling in context. Nat. Rev. Mol. Cell Biol. 13:616–630. 22992590 10.1038/nrm3434 PMC4027049

[phy213977-bib-0024] Melis, N. , M. Tauc , M. Cougnon , S. Bendahhou , S. Giuliano , I. Rubera , et al. 2014 Revisiting CFTR inhibition: a comparative study of CFTRinh‐172 and GlyH‐101 inhibitors. Br. J. Pharmacol. 171:3716–3727. Published online 2014 Jul 17. doi: [10.1111/bph.12726] 24758416 PMC4128068

[phy213977-bib-0025] Nicola, T. , J. S. Hagood , M. L. James , M. W. MacEwen , T. A. Williams , M. M. Hewitt , et al. 2009 Loss of Thy‐1 inhibits alveolar development in the newborn mouse lung. Am. J. Physiol. Lung Cell. Mol. Physiol. 296:L738–L750. 19270178 10.1152/ajplung.90603.2008 PMC2681351

[phy213977-bib-0026] Nicola, T. , N. Ambalavanan , W. Zhang , M. L. James , V. Rehan , B. Halloran , et al. 2011 Hypoxia‐induced inhibition of lung development is attenuated by the peroxisome proliferator‐activated receptor‐gamma agonist rosiglitazone. Am. J. Physiol. Lung Cell. Mol. Physiol. 301:L125–L134. 21531777 10.1152/ajplung.00074.2011 PMC3129902

[phy213977-bib-0027] Olave, N. , T. Nicola , W. Zhang , A. Bulger , M. James , S. Oparil , et al. 2012 Transforming growth factor‐beta regulates endothelin‐1 signaling in the newborn mouse lung during hypoxia exposure. Am. J. Physiol. Lung Cell. Mol. Physiol. 302:L857–L865. 22287612 10.1152/ajplung.00258.2011 PMC3362161

[phy213977-bib-0028] Paauwe, M. , R. C. Heijkants , C. H. Oudt , G. W. van Pelt , C. Cui , C. P. Theuer , et al. 2016 Endoglin targeting inhibits tumor angiogenesis and metastatic spread in breast cancer. Oncogene 35:4069–4079. 26804178 10.1038/onc.2015.509

[phy213977-bib-0029] Peterson‐Carmichael, S. L. , W. T. Harris , R. Goel , T. L. Noah , R. Johnson , M. W. Leigh , et al. 2009 Association of lower airway inflammation with physiologic findings in young children with cystic fibrosis. Pediatr. Pulmonol. 44:503–511. 19382221 10.1002/ppul.21044

[phy213977-bib-0030] Prieto, M. , A. B. Rodriguez‐Pena , A. Duwel , J. V. Rivas , N. Docherty , F. Perez‐Barriocanal , et al. 2005 Temporal changes in renal endoglin and TGF‐beta1 expression following ureteral obstruction in rats. J. Physiol. Biochem. 61:457–467. 16440600 10.1007/BF03168452

[phy213977-bib-0031] Rath, T. , L. Hage , M. Kugler , K. M. Menendez , R. Zachoval , L. Naehrlich , et al. 2013 Serum proteome profiling identifies novel and powerful markers of cystic fibrosis liver disease. PLoS ONE 8:e58955. 23516586 10.1371/journal.pone.0058955 PMC3597583

[phy213977-bib-0032] Rodriguez‐Pena, A. , M. Prieto , A. Duwel , J. V. Rivas , N. Eleno , F. Pérez‐Barriocanal , et al. 2001 Up‐regulation of endoglin, a TGF‐beta‐binding protein, in rats with experimental renal fibrosis induced by renal mass reduction. Nephrol. Dial. Transplant. 16(Suppl 1):34–39. 11369818 10.1093/ndt/16.suppl_1.34

[phy213977-bib-0033] Rodriguez‐Pena, A. , N. Eleno , A. Duwell , M. Arévalo , F. Barriocanal , O. Flores , et al. 2002 Endoglin upregulation during experimental renal interstitial fibrosis in mice. Hypertension 40:713–720. 12411467 10.1161/01.hyp.0000037429.73954.27

[phy213977-bib-0034] Rosen, L. S. , H. I. Hurwitz , M. K. Wong , J. Goldman , D. S. Mendelson , W. D. Figg , et al. 2012 A phase I first‐in‐human study of TRC105 (Anti‐Endoglin Antibody) in patients with advanced cancer. Clin. Cancer Res. 18:4820–4829. 22767667 10.1158/1078-0432.CCR-12-0098 PMC3432706

[phy213977-bib-0035] Rosen, L. S. , M. S. Gordon , F. Robert , and D. E. Matei . 2014 Endoglin for targeted cancer treatment. Curr. Oncol. Rep. 16:365. 24445497 10.1007/s11912-013-0365-x

[phy213977-bib-0036] Rowe, S. M. , S. Miller , and E. J. Sorscher . 2005 Cystic fibrosis. N. Engl. J. Med. 352:1992–2001. 15888700 10.1056/NEJMra043184

[phy213977-bib-0037] Shi, M. , J. Zhu , R. Wang , X. Chen , L. Mi , T. Walz , et al. 2011 Latent TGF‐beta structure and activation. Nature 474:343–349. 21677751 10.1038/nature10152 PMC4717672

[phy213977-bib-0038] Snodgrass, S. M. , K. M. Cihil , P. K. Cornuet , M. M. Myerburg , and A. Swiatecka‐Urban . 2013 Tgf‐beta1 inhibits Cftr biogenesis and prevents functional rescue of DeltaF508‐Cftr in primary differentiated human bronchial epithelial cells. PLoS ONE 8:e63167. 23671668 10.1371/journal.pone.0063167 PMC3650079

[phy213977-bib-0039] Stoltz, D. A. , D. K. Meyerholz , and M. J. Welsh . 2015 Origins of cystic fibrosis lung disease. N. Engl. J. Med. 372:1574–1575. 10.1056/NEJMc1502191 25875271

[phy213977-bib-0040] Sun, H. , W. T. Harris , S. Kortyka , K. Kotha , A. J. Ostmann , A. Rezayat , et al. 2014 Tgf‐beta downregulation of distinct chloride channels in cystic fibrosis‐affected epithelia. PLoS ONE 9:e106842. 25268501 10.1371/journal.pone.0106842 PMC4182049

[phy213977-bib-0041] Taddei, A. , C. Folli , O. Zegarra‐Moran , P. Fanen , A. S. Verkman , and L. J. Galietta . 2004 Altered channel gating mechanism for CFTR inhibition by a high‐affinity thiazolidinone blocker. FEBS Lett. 558:52–56. 14759515 10.1016/S0014-5793(04)00011-0

[phy213977-bib-0042] Velasco, S. , P. Alvarez‐Munoz , M. Pericacho , P. ten Dijke , C. Bernabéu , J. M. López‐Novoa , et al. 2008 L‐ and S‐endoglin differentially modulate TGFbeta1 signaling mediated by ALK1 and ALK5 in L6E9 myoblasts. J. Cell Sci. 121(Pt 6):913–919. 18303046 10.1242/jcs.023283

